# Supplementary figures and images for: Identification of Tumor Antigens and Immune Subtypes of Malignant Mesothelioma for mRNA Vaccine Development
Source: Vaccines (Basel). 2022 Jul 22;10(8):1168. doi: 10.3390/vaccines10081168 (PMC9331978; doi:10.3390/vaccines10081168)

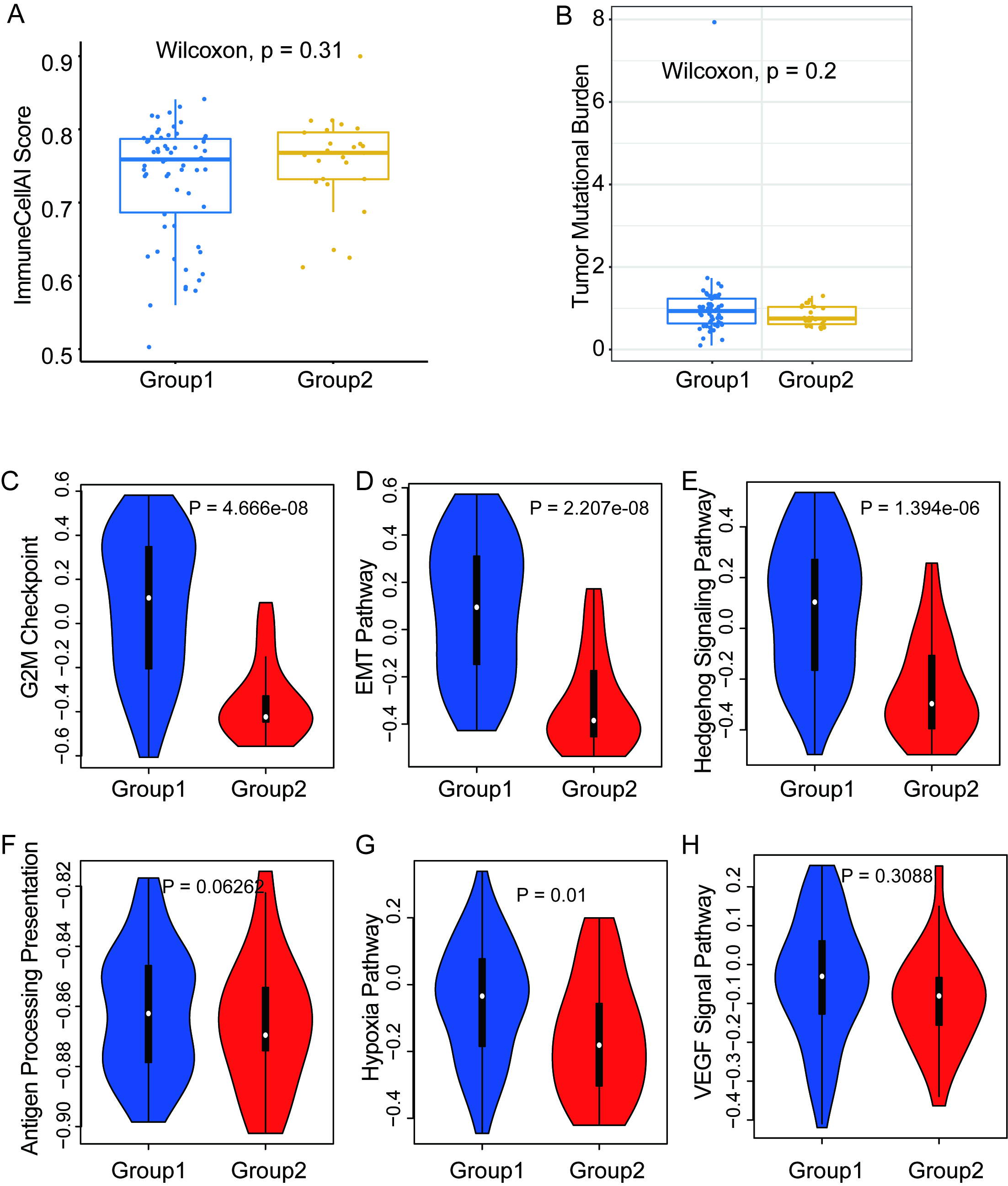

Supplement: Supplementary file 1 [file vaccines-10-01168-s001.zip › Supplementary Figure S1.tif]
